# Cloning and characterization of a new β-Glucosidase from a metagenomic library of Rumen of cattle feeding with *Miscanthus sinensis*

**DOI:** 10.1186/1472-6750-14-85

**Published:** 2014-10-02

**Authors:** Yadan Li, Ning Liu, Hui Yang, Fei Zhao, Ye Yu, Yun Tian, Xiangyang Lu

**Affiliations:** College of Bioscience and Biotechnology, Hunan Agricultural University, Changsha, 410128 China; Hunan Agricultural Bioengineering Research Institute, Changsha, 410128 China; College of Life Science, Hunan Normal University, Changsha, 410181 China; Departments of Physiology, Biochemistry, and Molecular Cell Biology, Institute of Medical Science, Shanghai Jiao Tong University School of Medicine, Shanghai, 200025 China

**Keywords:** β-glucosidase, Rumen, *Miscanthus sinensis*, Metagenomic library

## Abstract

**Background:**

The study on the second generation bio-fuel is a hot area of current research of renewable energy. Among series of key points in this area, the role of β-glucosidase in the degradation of intermediate gluco-oligosaccharides limits the rate of the complete saccharification of lignocellulose.

**Results:**

In this study, a new β-glucosidase gene, *unglu135B12*, which was isolated from a metagenomic library of rumen of cattle feeding with *Miscanthus sinensis* by the function-based screening, encodes a 779 amino acid polypeptide that contains a catalytic domain belonging to glycoside hydrolase family 3 (GH3). It was recombinantly expressed, purified and biochemically characterized. The recombinant β-glucosidase, unglu135B12, displayed optimum enzymatic activity at pH 5.0 at 38°C, and showed the highest specific activity of 2.5 × 10^3^ U/mg under this optimal condition to p-nitrophenyl-β-D-glucopyranoside (pNPG), and its *Km* and *Vmax* values were 0.309 mmol/L and 7.292 μmol/min, respectively. In addition, the presence of Ca^2+^, K^+^, Na^+^ slightly improved β-glucosidase activity of unglu135B12 by about 5%, while about 10 ~ 85% loss of β-glucosidase activity was induced by addition of Mn^2+^, Fe^3+^, Zn^2+^, Cu^2+^. Interestingly, unglu135B12 was activated by glucose at the concentration lower than 40 mM.

**Conclusions:**

Our findings indicate that unglu135B12 is a new β-glucosidase derived from rumen of cattle, and it might be a potent candidate for saccharification of lignocellulose in industrial application.

**Electronic supplementary material:**

The online version of this article (doi:10.1186/1472-6750-14-85) contains supplementary material, which is available to authorized users.

## Background

The study on the second generation bio-fuel, which can be constantly derived from the waste of agriculture and forestry and the widespread distribution of non-food plants, is a hot area of current research of renewable energy [1, 2]. Among non-food plants, as the raw material of bio-fuel, *Miscanthus sinensis* has been focused on in recent years as an ideal bio-source plant. This fiber-rich plant is widely distributed around South East Asia, and it’s also a common specie in China [3]. However, the complexity of lignocellulose of its cell wall makes the plant very difficult to be degraded [4]. Thus, it is urgent to explore new strong functional enzymes to solve the key problem in degradation of this plant, and to discover an economical way for the production of renewable bio-fuel from *Miscanthus sinensis*.

The conversion of the most abundant part of fiber, cellulose, into bio-fuel will depend on the synergistical hydrolysis of enzymes at least including three main cellulases: endo-β-1,4-glucanase (EC3.2.1.4), cellobiohydrolase (EC3.2.1.91) and β-glucosidase (EC3.2.1.21) [5]. β-glucosidase is widely distributed in nature [6, 7], which catalyses the cleavage of the glycosidic bonds existing in disaccharides, oligosaccharides and alkyl- or aryl- β-glucosides [8]. Although β-glucosidase does not act on cellulose directly, it is of great importance in cellulose deconstruction by eliminating cellobiose inhibition on endoglucanases and exoglucanases, allowing the cellulolytic enzymes to function more efficiently [5, 9]. Therefore, the activity of β-glucosidase is considered as the rate-limiting factor in cellulose degradation.

The low efficiency of β-glucosidases discovered in previous studies may be responsible for the high cost of bio-fuel production from lignocellulosic biomass [10], identification of new efficient β-glucosidases is thus important and necessary. Ruminants carry out a foregut fermentation that digests plant polysaccharides materials by a complicated and efficient microbial process [11]. Therefore, the habitat in rumen represents a rich hotspot for exploring diverse functional enzymes which could be used in lignocellulose degradation. Considering the fact that more than 85% of ruminal microbes remains uncultured, metagenomic technology, avoiding from pure culture, has drawn more attention during the study of finding new cellulolytic enzymes from this special environment [12].

Metagenomic strategy had been successfully employed in identification of new biocatalytic enzymes encoding genes from the uncultured component of microbial communities from a variety of environmental samples [13, 14]. In general, metagenomic method involves the extraction of metagenomic DNA from uncultured samples, the construction of the metagenomic libraries and the screening for the aimed genes [13]. This culture-independent method is able to avoid the limitation of the pure cultivation method, and many new β-glucosidases encoding genes have been obtained by means of metagenomics [15–17].

It had been demonstrated that the ratio of cellolytic bacteria was increased in large intenstine of *Miscanthus sinensis* feeding cattle [18]. Meanwhile, our unpublished result showed that the ratios of cellolytic bacteria and fungi in rumen were also increased due to the sole forage of *Miscanthus sinensis*. Therefore, exploring new cellolytic enzymes encoding gene from *Miscanthus sinensis* feeding cattle exhibit more potentiality. The aim of the present study was to identify new gene encoding β-glucosidase from total DNA isolated from ruminal microbes of Xiangxi yellow cattle fed on *Miscanthus sinensis* using the metagenomic techniques. Through function-based screening of the metagenomic library, one new β-glucosidase gene was obtained. The recombinant enzyme was overexpressed in Escherichia coli BL21(DE3) and investigated for the enzymatic properties.

## Results

### Construction and evaluation of the matagenomic library from microbes in Xiangxi yellow cattle rumen

**Figure 1:**
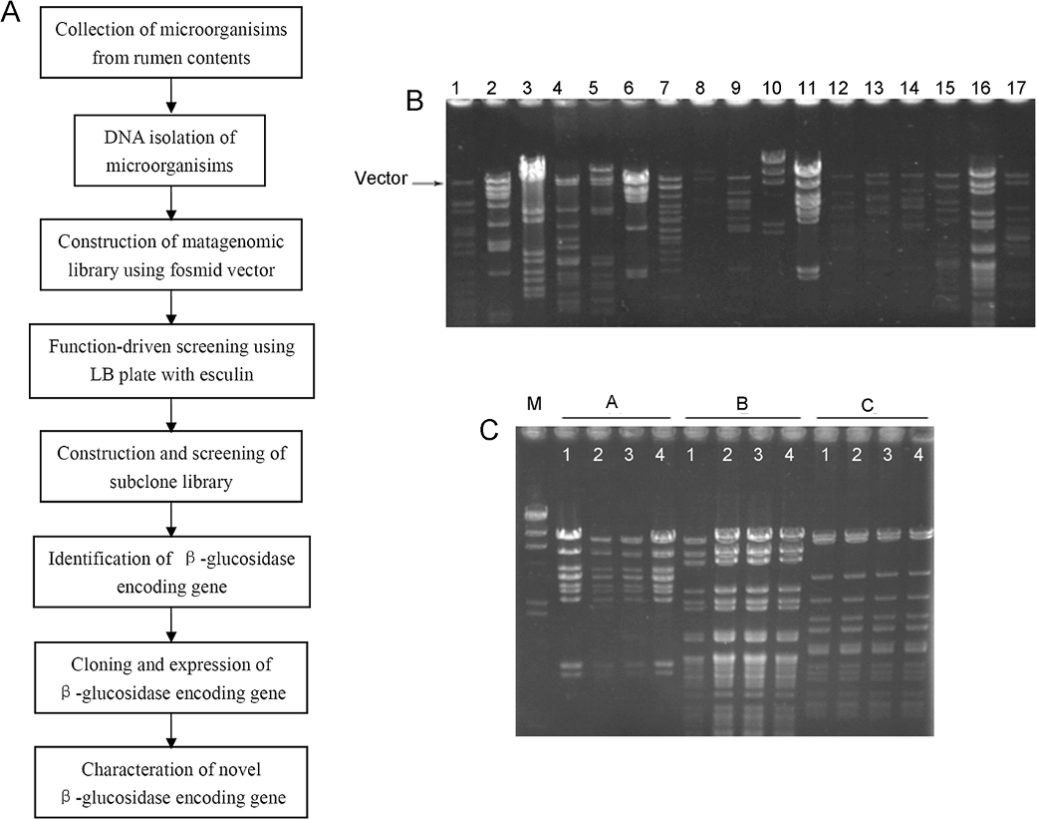
**Identification of a positive β-glucosidase functional clone 135B12 from metagenomic library of** ***Miscanthus sinensis*** **fed cattle’s ruminal inhabitants. A**. Strategy of obtaining of new β-glucosidase from metagenomic library. **B**. The volume of metagenomic library. **C**. Stability of exogenous gene in the library after subcultures (**A**, **B**, **C**: digestive pattern of fosmid DNA of three indipendent clones from the library by *EcoR* I and *Hind* III; 1-4: mean four times of subcultures).

To identify new β-glucosidase from metagenomic library, the strategy was described in Figure 1A. Rumen content samples were collected from Xiangxi yellow cattle in Hunan province. A fosmid library was constructed with metagenomic DNA isolated from microbes of rumen samples, containing 20,160 clones. The average insert size of clones was about 30 kb, and the full size of the library was estimated to be 600 Mb (Figure 1B). The stability of this metagenomic library was reliable according to the result of restriction endonuclease analysis that the insert fragments of three clones which were chosen randomly from the library were in accord with the original one after four times of subcultures (Figure 1C). Furthermore, the reliability of the present metagenomic library was also proved by the results of random sequencing of 92 clones’ insert fragments (Additional file 1: Table S1). One singlet of all had ≥97% similarity with *Bacteroides thetaiotaomicron* and two singlets had ≥97% similarity with uncultured *Bacteria*, which demonstrated the present metagenomic library was valid, while, other eighty-nine singlets didn’t have high homology with known sequences which perfectly confirmed that the library possessed great novelty (Additional file 1: Table S1).

### Annotation of β-glucosidase gene unglu135B12

**Figure 2:**
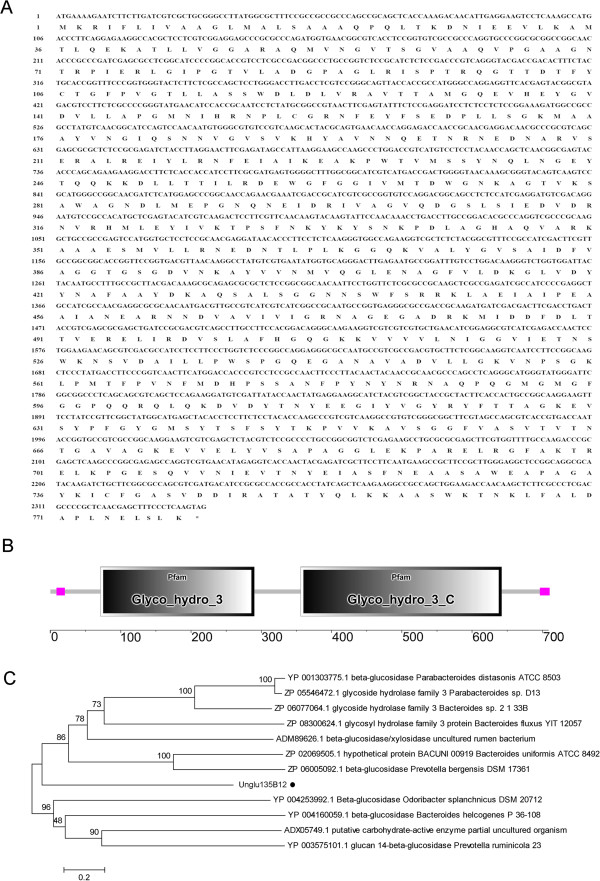
**Characterization of amino acid sequence of Unglu135B12. A**. amino acid sequence of Unglu135B12. **B**. Predicted modular architecture of Unglu135B12. **C**. Phylogenetic relationship of Unglu135B12 with related proteins. The scale bar corresponds to a genetic distance of 0.2 substitutions.

All of the clones were transferred to the screening plates for β-glucosidase activity. A clone expressing relatively strong β-glucosidase activity was obtained, named 135B12. Due to the average length of insert sequence of 135B12 was about 32 kb, sequence of the new gene encoding β-glucosidase was not fit for direct location. Thus subclone library was further constructed using the pUC19 vector to yield smaller fragments. After the screening by the same activity-based method as mentioned above, the positive subclone was sequenced. The length of predicted ORF was 2340 bp encoding a protein of 779 amino acids (Figure 2A). The new sequence had been submitted to NCBI, and the genebank access number was JX962691. The sequence of 2340 bp was shortly named as *Unglu135B12*, and the encoded protein was Unglu135B12 accordingly.

### Analyses of predicted β-glucosidase sequence and domain structures

The theoretical molecular mass of Unglu135B12 is 84.0 kDa, and the value of pI is 6.0 according to the analysis of DNAStar. Sequence analysis with SMART indicated that the putative protein has one glycosyl hydrolase family 3 domain (from 71 to 290 amino acid) and one glycosyl hydrolase family 3 C terminal domain (from 356 to 642 amino acid) (Figure 2B). Additionally, conserved domains search on NCBI also revealed that Unglu135B12 comprised these two conserved domains (figure didn’t show in this paper). Thus Unglu135B12 was probably a GH3 β-glucosidase. Information of phylogenetic tree showed it exhibited from 57% to 62% sequence identities to homologous proteins derived from bacteria of Bacteroidetes phylum (e.g. *Prevotella bergensis*) (Figure 2C).

### Expression and characterization of Unglu135B12

**Figure 3:**
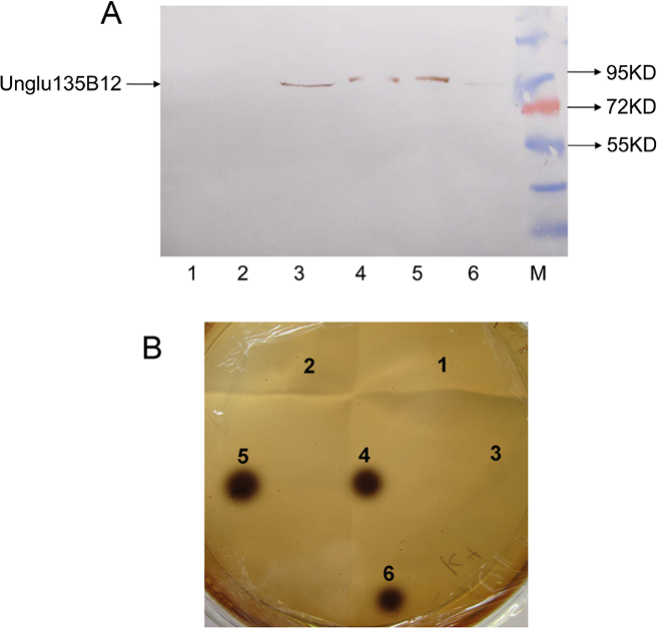
**Characterization of Unglu135B12. A**. Characterization of Unglu135B12 by western blot. *Lane* 1. Crude extract of BL21 (DE3) carrying pET28a (+) vector; *Lane* 2: IPTG-uninduced crude extract of BL21 (DE3) carrying pET-Unglu135B12; *Lane* 3: IPTG-induced crude extract of BL21 (DE3) carrying pET-Unglu135B12; *Lane* 4: Eluted Unglu135B12 protein after purification by Ni-NTA His · Bind Resins; *Lane* 5: Dialyzed Unglu135B12 protein; *Lane* 6: Flow through; *Lane* M: protein Marker. **B**. Functional verification of purified Unglu135B12. 1. Crude extract of BL21 (DE3); 2: Crude extract of BL21 (DE3) carrying pET28a (+) vector; 3: IPTG-uninduced crude extract of BL21 (DE3) carrying pET-Unglu135B12; 4: IPTG-induced crude extract of BL21 (DE3) carrying pET-Unglu135B12; 5: Eluted Unglu135B12 protein after purification by Ni-NTA His · Bind Resins; 6: Dialyzed Unglu135B12 protein.

In order to characterize Unglu135B12, *unglu135B12* was cloned into pET28a (+) and heterologously expressed in BL21 and purified. The western bolt detection showed the positions of expressed protein and purified protein, which were in accordance with the predicted MW value (Figure 3A). Furthermore, the purified Unglu135B12 was tested on the functional screening plate and it was demonstrated that the heterologous protein before/after purification and dialyzed protein were still with the β-glucosidase activity (Figure 3B).

**Figure 4:**
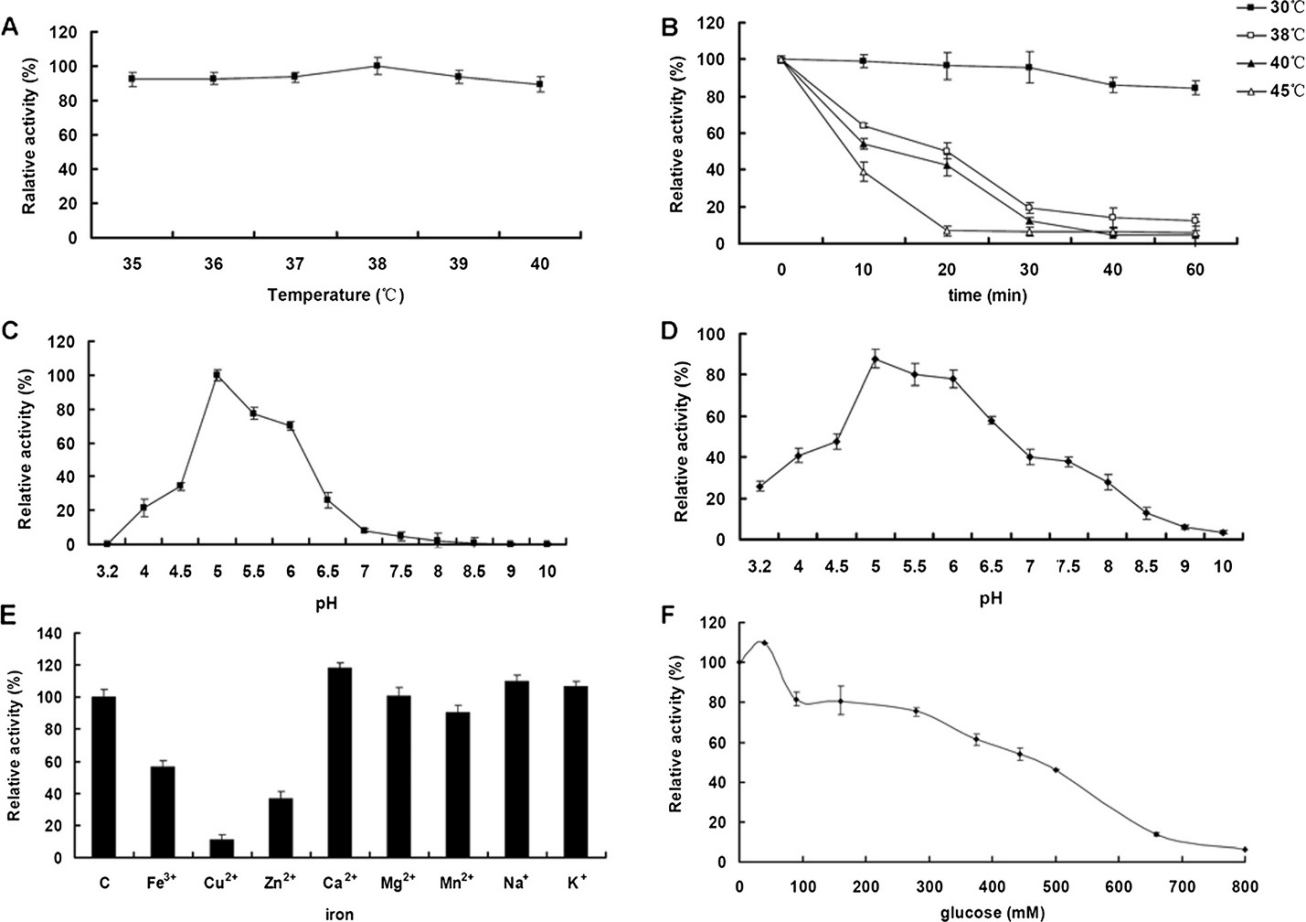
**Effect of pH and temperature on the enzyme activity and stability of unglu135B12. A**. Effect of temperature on the activity of unglu135B12. **B**. Effect of pH on the activity of unglu135B12. **C**. Temperature stability of unglu135B12. **D**. pH stability of unglu135B12. **E**. Effect of metal ions on activity of unglu135B12. **F**. Influence of glucose on enzyme activity with pNPG as the substrate. Enzymatic reactions without glucose were used as controls. All measurements were analysed in triplicate and error bars were indicated.

To determine enzymatic characteristics of this purified putative GH3 β-glucosidase, pNPG was used as substrate for testing the activity of β-glucosidase. As shown in the present data, the optimal temperature was 38°C and the optical pH was 5.0 (Figure 4A and C), with the specific activity of 2.5 × 10^3^ U/mg. The relative activity of Unglu135B12 still reserved 60% when placed it under pH5.0-6.0 at 4°C for 24 h. The stability features of Unglu135B12 were not very satisfied, as it was stable only in a narrow range of a battery of temperatures and pHs (Figure 4B and D).

The addition of Ca^2+^, K^+^, Na^+^ slightly enhanced β-glucosidase activity of Unglu135B12 by about 5% and about 10-85% loss of β-glucosidase activity was induced by addition of Mn^2+^, Fe^3+^, Zn^2+^, Cu^2+^. Mg^2+^ had little effect on activity of Unglu135B12 (Figure 4E).

The activity of Unglu135B12 towards pNPG was enhanced by glucose at concentrations lower than 40 mM (Figure 4F). In the presence of 40 mM glucose, the activity of Unglu135B12 increased to a maximum value with 9.6% more than that of the control without glucose. With glucose further increasing, the enzyme activity was gradually inhibited.

The reaction kinetic parameters of the purified enzyme were measured from double reciprocal Lineweaver-Burk plots. This putative β-glucosidase had a *K*_*m*_ value of 0.309 mmol/L, a *Vmax* value 7.292 μmol/min.

### Analysis of 3D structure and substrate docking

**Figure 5:**
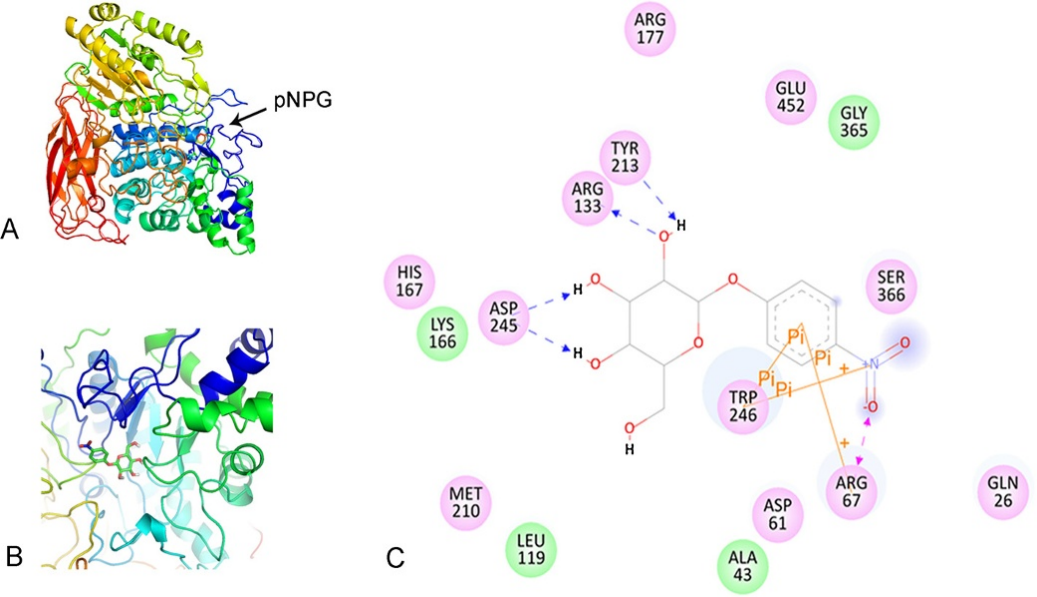
**Structural models of β-glucosidases Unglu135B12 characterised in the present work and its substrate docking. A**. The pNPG-binding model of Unglu135B12 derived from *Thermotoga neapolitana* β-glucosidase 3B (PDB ID 2X40). The three protein domains: α/β barrel, α/β sandwich and fibronectin-like, were coloured cyan, kelly and red, respectively. **B**. Closeup of the catalytic centre. **C**. The key residues in the catalytic pocket around pNPG.

Based on structural analysis, Unglu135B12 was composed by three domains: an α/β barrel, an α/β sandwich and a fibronectin-like domain at carboxyl-terminal (Figure 5A). It was supposed that α/β barrel and α/β sandwich were able to contribute each one of the two catalytic residues, while, the function of fibronectin-like domain was still unclearly and this domain was not present in all enzyme of GH3 family [19]. Molecular docking was conducted with the substrate pNPG by the tool of Discovery Studio LibDock based on this lowest-energy model. In the light of the need of clearer description of the relative location between enzyme and substrate, then it was amplified appropriately as Figure 5B. Moreover, Figure 5C indicated that some key residues existed around pNPG in the model, which could probably form hydrogen bonds and phosphoanhydride bonds and played irreplaceable roles in the catalytic pocket (Figure 5C).

## Discussion

In this present work, we used *Miscanthus sinensis*, a promising cellulosic energy crop, as the plant substrate for feeding experimental animal. To explore new β-glucosidase gene from the microbial communities of these *Miscanthus sinensis* fed cattle, a metagenomic library containing about 20,160 clones was constructed, which was screened by function-based method. Consequently, the gene *Unglu135B12* was identified as the potential candidate gene. The purified protein Unglu135B12 showed high specific activity to the substrate pNPG, and its activity could be influenced by different irons. Besides, the inhibition of glucose on Unglu135B12 began to appear only when the concentration of glucose was more than 40 mM. These data provided strong evidence to support the feasibility of searching functional enzyme for mono-/polysaccharides degradation from rumen habitat. Rumen habitats harbor a vast microbial diversity, including various anaerobic bacteria, archaea and fungi that are different from those found in water, soil or other environments. This special microbial community is closely related to the fiber-rich diet, due to the synergistic actions of cellulase, hemicellulase, ligninolytic enzymes and β-glucosidase derived from ruminal microbes, and fullfills the degradation of complex structure of plant in animal’s diet [4, 20]. Therefore, the metagenomic library in our work distinguished itself as an ideal extensive resource for searching cellulolytic enzymes or other enzymes related to this degradation process.

On the basis of phylogenetic analysis, Unglu135B12 was mostly closed to one beta-glucosidase from *Prevotella bergensis*, belonging to Bacteroidetes (Figure 2C). This indication was in line with the results of our unpublished studies, in which we comparatively analyzed the phylogenetic relationship of ruminal bacteria between *Miscanthus sinensis* fed cattle and mixed forage fed cattle and found that the proportion of Bacteroidetes was increased significantly induced by the sole diet of *Miscanthus sinensis*. So it emphasized again the relevance between feeding of *Miscanthus sinensis* and rise trend of Bacteroidetes in rumen. With the function of glycosyl hydrolase, Bacteroidetes play key roles in the decomposition of carbohydrate polymers and proteins in digestive system of mammal [21, 22]. In the light of this point, it’s worthy to further detect new glycosyl hydrolase from the present microbial metagenomics library.

In addition, it was said that microbial genomes often contain a substantial number of glycosyl hydrolase (GH) genes, many of which could be induced by different carbon sources. The evidence from conserved domain analysis strongly indicated that Unglu135B12 was a member of glycosyl hydrolase family 3 (GH3). GH3 is one of the largest GH families with more than 4800 sequences in the CAZy database (http://www.cazy.org) and the relative abundance and distribution of GH3 in the metagenome from the bovine rumen microbiome is 18.4% [23].

After the western blot analysis using the specific anti-his tag antibody, the molecular mass of the rumen microbial protein was recognized as about 85 kD, in consistent with the predicted value, which suggested that this protein was probably a monomeric protein. Moreover, the purified β-glucosidase Unglu135B12 from the heterologous expression system were identified that it efficiently hydrolyzed pNPG within a narrow pH range of 5.0-6.0, and had optimal activity at 38°C, which indicated the native Unglu135B12 protein might play an active role under *in vivo* conditions in the cattle rumen. However, these features of the recombinant protein might hinder its industrial application. Given the limitation of Unglu135B12 in industrial production, it’s worthy to improve its stability of pH and temperature by biotechnological methods such as site-directed mutagenesis, codon randomisation [24] or immobilization techniques [25]. Therefore, with the help of the prediction by homology modeling and molecular docking, site-directed mutagenesis against the key residues could be considered as a new orientation in further research. While, the specific activity of Unglu135B12 against pNPG was measured as 2.5 × 10^3^ U/mg at pH 5.0 when assayed at 38°C. It was considered as a relatively high value compared with other β-glucosidases found in uncultured microbes or pure-isolated microbes [15, 26]. Besides, addition of Ca^2+^, K^+^, Na^+^ could enhance its activity to 105%. Based on this information, it’s hopefully to achieve more enhancement of Unglu135B12’s activity. Furthermore, most of the microbial β-glucosidases reported to date are competitively inhibited by glucose, while several β-glucosidases from a few fungi and yeasts show high glucose tolerance [27, 28]. Unglu135B12 is activated by glucose at concentrations below 40 mM and remains more than 75% activity with glucose at concentrations of 280 mM. In further study, the tolerance to glucose of unglu135B12 could be probably improved by protein engineering [29].

Overall, the reason why unglu135B12 exhibited these characteristics described above could be attributed to its source. Consistent with other β-glucosidases obtained by metagenome method from herbivore’s digestive tract, their optimal pH and temperature were in a narrow range which similar to their natural environment [15, 26]. In addition, it’s worth mentioning that the activity of unglu135B12 is significantly higher than that of the β-glucosidases from rabbit cecum [26] and yak rumen [15], moreover, the unglu135B12 still showed tolerance to glucose. These points might indicate more potentiality of further directed mutagenesis on unglu135B12.

## Conclusions

The more effective β-glucosidase could definitely convert cellobiose into glucose more conveniently and economically, and the combination of this new β-glucosidase and cellulase or hemicellulase will be beneficial for break the major bottleneck in industrial-scale conversion of cellulosic biomass into biofuels. Furthermore, discovery of other high preformance new monofunctional enzyme or multifuctional enzyme from our library is hopefully believed to provide more possibility for their applications in future industrial production. Besides, heterologously expressed functional enzyme by genetically modifying host organism could also be an effective tool in developing enzyme with desired properties.

## Methods

### Animal ethics

Care of laboratory animals and animal experimentation were performed in accordance with animal ethics guidelines and approved protocols. All animal experiments were performed according to the Hunan Community Rules of Animal Care with the permission number 38-61 of Hunan Agricultural University Veterinary Services (China).

### Metagenomic fosmid library construction and evaluation

Matagenomic DNA was extracted from rumen content of Xiangxi yellow cattle which were fed with sole fresh *Miscanthus sinensis* from 2.5 years old for 18 months. The pooled samples were collected and stored at -80°C until DNA extraction.

Total DNA was extracted from the pooled samples as described before [18]. DNA fragments of about 36 ~ 48 kb were recovered from gel by electroelution for the library construction by using pCC2FOS™ Vector (Epicentre, USA). All of operation was following the manufacturer’s instruction. Library clones were duplicated and stored in 96-well plates at -80°C.

Clones of fosmid library were cultured in 96-well plates containing 70 μl lysogeny broth (LB) supplemented with chloramphenicol (22.5 μg/ml) at 37°C overnight. Then LB plates supplemented with esculin hydrate (0.1%), ferric ammonium citrate (0.25%) and chloramphenicol (22.5 μg/ml) were used for screening functional clones with β-glucosidase activity according to the method described by Eberhart et al [30]. Positive clones were detected by the formation of a black halo around the colonies after incubation at 37°C for 20-24 h. The strength of enzyme activity was initially decided by comparing the size of each black halo.

To evaluate the volume and stability of this library, we conducted the analysis of restriction endonuclease map with *EcoR* I and *Hind* III. Moreover, reliability of this library was detected by random sequencing.

### Subclone and sequence analysis

Due to the length of insert sequence in each clone of fosmid library was about 40 kb, it was too long to directly sequence the gene of β-glucosidase from positive clone. Therefore, Fosmid DNA of positive clone was extracted and digested by *EcoR* I and *Hind* III. Then DNA fragments around 1 ~ 5 kb length were ligated into the vector pUC19 (Promega, USA), and transformed into *E. coli* DH5α. After screening by the same method mentioned before, a positive subclone was detected and sequenced.

The open reading frames (ORF) of the aimed sequence were analyzed by DNAMAN. The domain architecture and conserved domain was analyzed by the online program SMART (http://smart.embl-heidelberg.de). The phylogenetic tree was constructed with MEGA4.0 software. Reliability of phylogenetic reconstruction was estimated by Boot-strapping values (1,000 replicates).

### Gene expression and protein purification

*Unglu135B12* was amplified by PCR using the primers 135B12F (5′-CGGAATTC TCCTTTGTCTACCTTTGTCAG-3′) containing a *EcoR* I site (underlined) and 135B12R (5′-ATGCGGCCGCCGAGGTATTTACAAAACACG-3′) containing a *Not* I site (underlined), then the PCR fragments were digested with restriction endonucleases *EcoR* I and *Not* I and cloned into pET28a (+) digested with the same enzymes, resulting in the recombinant plasmid pET28a/Unglu135B12. For expression of Unglu135B12, pET28a/Unglu135B12 was transformed into *E coli* BL21 (DE3), then was induced with 0.1 mmol/L IPTG at 25°C for 4.5 h. Ni-NTA resin was used to purify the protein, and then dialyzed the protein with 20 mM PBS buffer (pH7.0) overnight at 4°C. The protein before/after purification, flow-through and dialyzed fusion protein were analyzed by SDS-PAGE and western blot.

All protein samples (15 μg) were separated by 8% SDS-PAGE and transferred onto a PVDF membrane (Bio-Rad). Then anti-His Tag (6×) antibody was used to performed the immunoblots. The specifically stained bands were clearly visible by DAB staining.

### Enzyme assays and characterization of Unglu135B12

The specific activity measurement was preformed as described by Feng [26], and one unit of β-glucosidase activity was defined as the amount of enzyme releasing 1 μmol p-nitrophenol from p-nitrophenyl-β-glucopyranoside (pNPG). The amount of released p-nitrophenol was measured by the absorbance at 405 nm [31].

The temperature optimum for the freshly purified β-glucosidase activity was measured in a range from 25°C to 65°C by incubating Unglu135B12 at pH 5.0. The effect of pH on enzyme activity was evaluated at pHs ranged from 3.2 to 10 at optimal temperature. The thermostability of Unglu135B12 was evaluated by incubating the recombinant enzyme at 30°C, 40°C, 45°C and optimal temperature at pH 5.5 for 1 h, and then measuring the residual activity. The stability of Unglu135B12 against pH was assayed by measuring the residual activity of Unglu135B12 in the buffer at different pHs after incubation at 4°C for 24 h.

Various cations (3.5 mM) were used to investigate the effects of metal ions on the β-glucosidase. Effect of glucose on the β-glucosidase activity was also evaluated, which was studied by determining the activity of β-glucosidase towards pNPG in presence of dose-dependent glucose (0 mM, 40 mM, 90 mM, 160 mM, 280 mM, 375 mM, 444 mM, 500 mM, 660 mM, 800 mM).

To determine the *K*_*m*_ and *Vmax* values, reactions were acted under optimal condition with pNPG ranging from 0 to 8 mmol l^-1^ according to the double-reciprocal plot method. All measurements were analyzed in triplicate.

### Homology modeling and substrate docking

The translation product of aimed sequence *unglu135B12* was further analyzed for protein domains using the Pfam-A database [32]. Model of β-glucosidases unglu135B12 were obtained from the SWISS-MODEL server (http://swissmodel.expasy.org/) using the coordinates of known β-glucosidase structure (PDB ID 2X40: β-glucosidase 3B from *Thermotoga neapolitana*) as the template. The lowest-energy model was chosen for the docking of the substrate of pNPG using Discovery Studio LibDock.

## Electronic supplementary material

Additional file 1: Table S1: The result of random sequencing. (PDF 25 KB)
