# Supplementary material for: Cloning and characterization of a new β-Glucosidase from a metagenomic library of Rumen of cattle feeding with Miscanthus sinensis
Source: BMC Biotechnol. 2014 Oct 2;14:85. doi: 10.1186/1472-6750-14-85 (PMC4287584; doi:10.1186/1472-6750-14-85)
Supplement: Supplementary file 1 — Additional file 1: Table S1: The result of random sequencing. (PDF 25 KB) [file 12896_2014_961_MOESM1_ESM.pdf]

Table S1 The result of random sequencing

singlet number	92
Average sequence lenghth	656 bp
Results of sequences blast in NCBI	1 singlet had ⩾97% similarity with *Bacteroides thetaiotaomicron* (1.08% of all sequenced singlets).<br>2 singlets had ⩾97% similarity with uncultured *Bacteria* (2.16% of all sequenced singlets).<br>89 singlets didn't have high similarity with known sequences (96.76% of all sequenced singlets).
